# Endoscopic Management of a Long-Duration Esophageal Food Impaction: A Case Report

**DOI:** 10.7759/cureus.58829

**Published:** 2024-04-23

**Authors:** Santiago Philibert-Rosas, Israel Podolsky Rapoport

**Affiliations:** 1 Health Sciences, Anahuac University, Mexico City, MEX; 2 Gastroenterology and Gastrointestinal Endoscopy, ABC Medical Center, Mexico City, MEX

**Keywords:** digestive endoscopy, esophagus, gastrointestinal endoscopy, food impaction, upper endoscopy

## Abstract

Foreign object ingestion (FOI) is a potentially life-threatening pathology that affects all ages, from children to older adults. The classification includes true FOI and esophageal food impaction (EFI), and each presents unique challenges. Endoscopic intervention is often required to prevent complications. Flexible endoscopes are the preferred management tool, ensuring a high success rate and safety. The following text presents a case of a 48-year-old male with a 5-day undiagnosed esophageal food impaction and the approach taken.

## Introduction

Foreign body ingestion poses a global challenge, especially among children, but adults with certain conditions are also vulnerable. Despite most cases passing without incident, 10-20% necessitate endoscopic intervention to prevent severe complications like impaction, ulceration, and perforation [1]. It comprises two main categories: true foreign object ingestion (FOI) and esophageal food impaction (EFI), each posing unique challenges. FOI involves items like bones and coins, often seen in children. At the same time, EFI, commonly caused by meat, is more prevalent in the United States and associated with esophageal disorders such as Zenker diverticulum and Schatzki rings [2-4]. The following text presents a case of a 48-year-old male experiencing postprandial vomiting after consuming steak. Upper endoscopy revealed impacted meat in the lower esophagus, requiring multiple maneuvers for successful removal and clearance. 

## Case presentation

A 48-year-old Hispanic male arrives at the clinic with complaints of postprandial vomiting following the consumption of solid food. He reported eating a steak five days prior to his visit and denied experiencing any relevant pain symptoms during questioning. He mentioned that before coming to the clinic, he had sought treatment at an outside health facility where he received IV fluids and an antispasmodic medication but experienced no relief from his symptoms. Before the diagnostic study, the patient was instructed to ingest small amounts of water, during which he reported no vomiting or other symptoms. Suspecting food impaction, an upper endoscopy was performed. 

**Figure 1:**
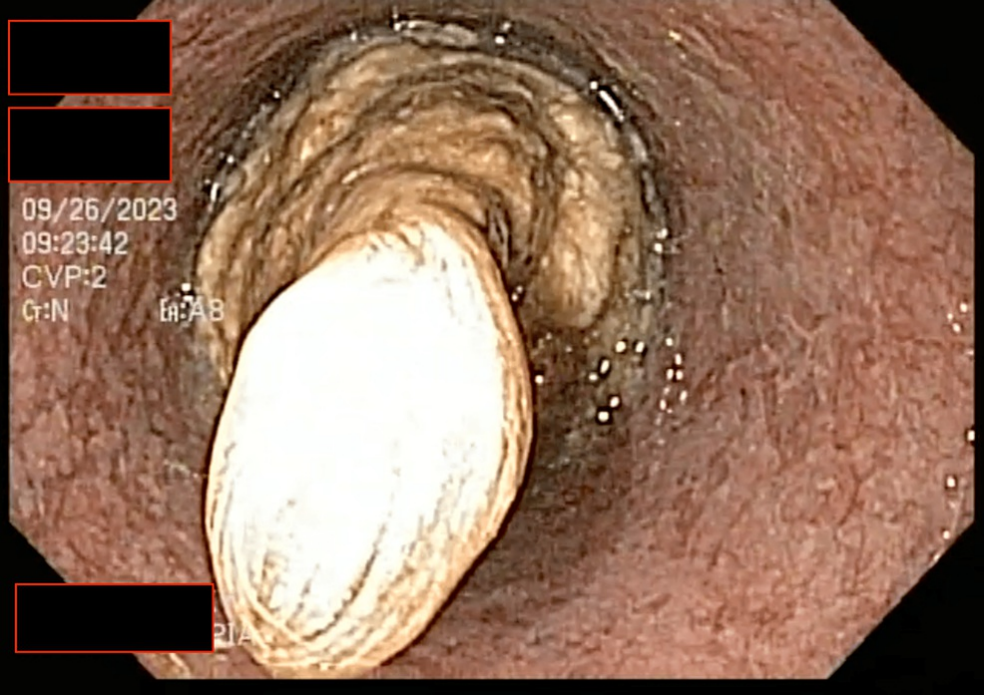
Impacted bolus. Food bolus obstruction in the esophagus, as viewed through the endoscope.

Esophagogastroduodenoscopy revealed impacted meat covering the entire diameter of the lower third of the esophagus (Figure 1). Multiple attempts were made to push the bolus gently into the stomach using the endoscope, but they proved unsuccessful. Polyp forceps were repeatedly employed to reposition the impacted bolus and facilitate its passage into the stomach. However, the soft and shredded bolus composition made repositioning it impossible. Nevertheless, breaking down the bolus into smaller fragments created an opening within the esophagus.

**Figure 2:**
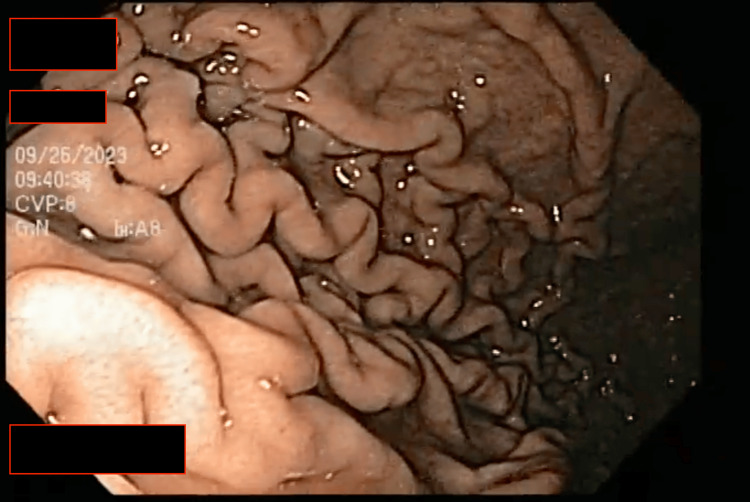
Stomach free of obstruction. As the endoscope enters the stomach, it reveals an empty stomach. The stomach lining appears devoid of any visible food particles or residue.

**Figure 3:**
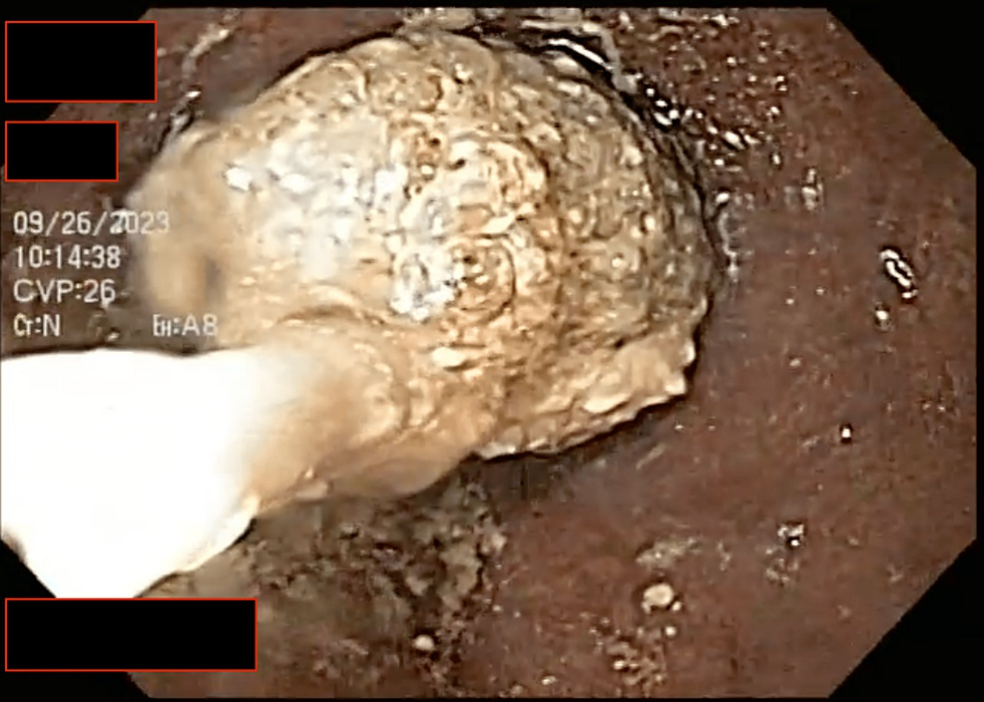
Net loop removing impaction. The net loop gently maneuvers around the impacted food bolus in the esophagus, delicately ensnaring and dislodging it from the obstructed passage.

**Figure 4:**
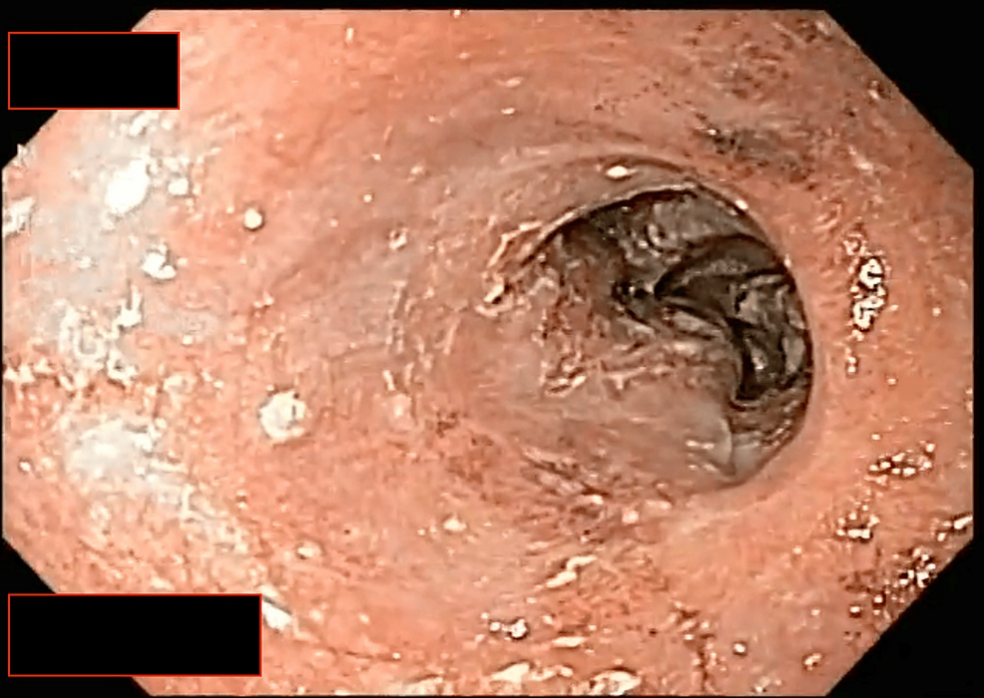
Unblocked esophagus. With precise and controlled movements, the net loop carefully extracts the obstruction, freeing the esophagus and restoring normal swallowing function.

This breach within the esophageal lumen allowed us to advance the endoscope into the stomach. Upon examination, we observed the absence of any remaining food, confirming that the bolus was confined to the esophagus (Figure 2). Multiple maneuvers were performed with the polyp forceps to fragment the bolus further, attempting to facilitate its passage into the stomach. With limited success, a net loop was employed to separate more significant portions of the bolus for removal through the oral cavity (Figure 3), with the primary goal of ensuring the airway remained clear of any potential complications. Ultimately, the remaining portion of the bolus was pushed into the stomach without resistance, resulting in the complete clearance of the esophagus (Figure 4). 

## Discussion

Foreign body ingestion poses a common and potentially life-threatening clinical challenge, with an estimated annual incidence of 13/100,000 cases worldwide and 120,000 annually in the United States [2,5]. Predominantly affecting children due to curiosity and accidental ingestion, adults with psychiatric disorders or seeking secondary gain are also at risk. While many ingested foreign bodies pass through the gastrointestinal tract without issue, 10-20% necessitate intervention, primarily endoscopic, to prevent complications such as impaction, ulceration, perforation, and potential death [1]. The classification includes true foreign object ingestion (FOI) and esophageal food impaction (EFI), each presenting unique challenges. True FOI encompasses bones, commonly from fish, and items like coins, batteries, and magnets, mainly observed in children. 

On the other hand, EFI predominantly involves incidents with meat, being the most common cause in the United States, setting it apart from true FOI [3-4]. Esophageal obstructions are most commonly encountered in the gastrointestinal tract, with the esophagus being the primary site. In most adult cases (88-97%), food impaction occurs at locations characterized by pathological or physiological luminal constriction. Underlying esophageal disorders, including Zenker diverticulum, Schatzki rings, peptic strictures, esophagitis, motor disorders, achalasia, radiation-induced stricture, and esophageal carcinoma, are frequently associated with food bolus impactions [1,2].

Optimal management for the majority of FOIs and EFIs involves the use of flexible endoscopes. This strategy boasts a high success rate, is typically safer than rigid endoscopy, and can be executed with moderate sedation in many cases [6-7]. Nowadays, various tools and accessories offer solutions for handling FOIs and EFIs. These include, but are not limited to, rat-tooth and alligator forceps, polypectomy snares, multi-prong graspers, Dormia baskets, Roth retrieval nets, Foley catheters, and variceal ligator caps, and more recently, balloon dilators and sutures have also been described as usable accessories in dealing with the presented scenario [1,8-9]. In endoscopic interventions, overtubes emerge as indispensable tools, crucial in handling FOI and EFI. Their utility is safeguarding the airway during retrieval, enabling multiple endoscope passes, and protecting the esophageal mucosa from potential harm when extracting sharp or pointed items. In cases where the object is positioned distally to the esophagus, it is advisable to use an extended overtube that spans the esophagogastric junction to add protection [1,10].

While an overtube enhances endoscopic procedures, it also brings inherent safety concerns; primary complications include mucosa scratches or tears, often arising from the large diameter and mucosa pinching between the scope and the overtube. Additionally, severe complications might include esophageal perforation, variceal rupture, and pneumomediastinum [11].

The decision not to employ an endoscopic overtube stemmed from the practical constraint of its unavailability during the procedure. Despite recognizing the potential benefits of an overtube, the team proceeded without one as it was not immediately accessible, prioritizing the timely execution of the intervention under the circumstances [1]. In the presented report, using an overtube can be considered unnecessary given that the EFI was easily manageable, there is a low risk of mucosal injury or complications during management, and the patient's airway was not compromised. Furthermore, the decision to forego the use of an overtube may be influenced by the size, shape, and nature of the object [6]. If the object poses a low risk of causing damage during extraction, the clinician might proceed without an overtube. It is essential to note that the medical team's expertise plays a pivotal role in determining whether an overtube is necessary [7,11]. Experienced endoscopists may feel confident in their ability to safely retrieve particular objects without the added protection of an overtube, taking into account the specific characteristics of each case.

## Conclusions

Foreign body ingestion presents a significant challenge, particularly in true foreign object ingestion and esophageal food impaction. Optimal management often involves endoscopic intervention, utilizing various tools and accessories tailored to the specific scenario. While endoscopic overtubes are valuable in certain situations, their necessity depends on factors such as the nature of the foreign body, the risk of mucosal injury, and the clinician's expertise. 

In the case report, the decision not to use an endoscopic overtube was influenced by practical constraints and the manageable nature of the esophageal food impaction. While overtubes offer additional protection, experienced endoscopists may opt to proceed without them in select cases, prioritizing timely intervention and considering the specific characteristics of the foreign body and patient presentation.
